# On Programmed Cell Death in *Plasmodium falciparum: Status Quo*

**DOI:** 10.1155/2012/646534

**Published:** 2012-01-12

**Authors:** Dewaldt Engelbrecht, Pierre Marcel Durand, Thérèsa Louise Coetzer

**Affiliations:** ^1^Plasmodium Molecular Research Unit, Department of Molecular Medicine and Haematology, School of Pathology, Faculty of Health Sciences, University of the Witwatersrand and National Health Laboratory Service, Johannesburg 2193, South Africa; ^2^Evolutionary Medicine Unit, Department of Molecular Medicine and Haematology, School of Pathology, Faculty of Health Sciences, University of the Witwatersrand and National Health Laboratory Service, Johannesburg 2193, South Africa; ^3^Department of Molecular Medicine and Haematology, Wits Medical School, Room 7Q11, 7 York Road, Parktown, Johannesburg 2193, South Africa

## Abstract

Conflicting arguments and results exist regarding the occurrence and phenotype of programmed cell death (PCD) in the malaria parasite *Plasmodium falciparum*. Inconsistencies relate mainly to the number and type of PCD markers assessed and the different methodologies used in the studies. In this paper, we provide a comprehensive overview of the current state of knowledge and empirical evidence for PCD in the intraerythrocytic stages of *P. falciparum*. We consider possible reasons for discrepancies in the data and offer suggestions towards more standardised investigation methods in this field. Furthermore, we present genomic evidence for PCD machinery in *P. falciparum*. We discuss the potential adaptive or nonadaptive role of PCD in the parasite life cycle and its possible exploitation in the development of novel drug targets. Lastly, we pose pertinent unanswered questions concerning the PCD phenomenon in *P. falciparum* to provide future direction.

## 1. Introduction

Programmed cell death (PCD) forms an integral physiological part of multicellular organisms, where it plays an essential role in normal development and maintenance of integrity and homeostasis. In addition, it forms part of the defense response to combat infectious pathogens as well as being involved in the pathogenesis of certain human diseases (reviewed in [1–3]). This self-sacrificial cell-death phenomenon has also been demonstrated in unicellular organisms, including parasitic protozoa (reviewed in [4, 5]). Apart from the ability to orchestrate their own death, parasites can facilitate their development and survival by inducing PCD in host cells (reviewed in [6]). These host-pathogen interactions are complex, and the role of PCD in balancing pathogenic and survival mechanisms remains poorly understood.

The definitions of PCD and its various phenotypes are considered in Table 2 of Appendix A. Observations of PCD have their foundations in the middle-late nineteenth century as an awareness of physiological cell death [7, 8] although the term was first coined in 1964 by Lockshin and Williams [9]. Apoptosis, now recognised as a prominent phenotype of PCD, was described in 1972 by Kerr and coworkers [10]. More than 20 years later, apoptosis was demonstrated in a unicellular trypanosome [11], and in 1997, it was described in two species of malaria parasites, *Plasmodium falciparum *[12] and *P. yoelii* [13]. Different phenotypes of PCD have been shown in evolutionary diverse unicellular eukaryote lineages [14, 15] as well as in prokaryotes [16]. However, a growing body of conflicting evidence regarding PCD in *Plasmodium* has followed. We present a critical review of current knowledge of this phenomenon, focusing on the asexual intraerythrocytic stage of *P. falciparum*, and offer possible explanations for discrepancies in the data. We also highlight some of the unanswered questions in this field, including the possible adaptive value of PCD, and allude to the possible exploitation of this process in the identification of novel drug targets.

## 2. Evidence of PCD in *Plasmodium falciparum*

The appearance of “crisis form” morphology, first described in *P. brasilianum* in 1944 [17], has been widely observed in *P. falciparum* and correlated with retardation of growth and development, loss of synchronicity, and decline in parasite numbers [18–22]. This morphological phenomenon was linked to PCD by Picot et al. [12]. Many studies have since cited the appearance of “crisis forms” as evidence of PCD [12, 23–27]. However, the definition of “crisis forms” is not entirely clear, often being simply described as degenerate or abnormal parasites, making it a parameter that is difficult to objectively observe and quantify. In addition, we and others have also observed the appearance of such degenerate parasites in untreated *in vitro *cultures ([12, 23], Engelbrecht et al., unpublished). Striving towards a unified description of cell death phenotypes, it has been suggested that morphological descriptions be replaced by functional and/or biochemical criteria [28].

Several PCD markers of an apoptosis-like phenotype have been documented in the ookinetes and zygotes of *P. berghei, * both *in vivo* in the *Anopheles* mosquito as well as in *in vitro* cultures, without external experimental stimuli [29–31] although Le Chat and colleagues found very little evidence to support this view [32]. Recently, it was shown that ookinetes of *P. falciparum* exhibited evidence of an apoptosis-like cell death in the midgut of the mosquito [33].

In the pathogenic asexual human blood stages of *P. falciparum,* the biochemical evidence for PCD and especially the phenotype of cell death remains highly controversial. Some studies support the occurrence of PCD as apoptosis [12, 26, 27, 34–36], while others suggest that the phenotype more closely resembles autophagic cell death [37] or necrosis [38]. Some overlap of apoptosis and autophagy has also been noted [24], while other authors simply describe the cell-death phenotype as nonapoptotic [25, 39]. At this stage, it cannot be conclusively confirmed whether any PCD phenotype is typical and whether its manifestation is essential and/or beneficial to the parasite. Resolution of these issues is an essential first step in elucidating the underlying PCD pathways in *P. falciparum* and their effect on host-pathogen interactions. A better understanding of both the proximate (“how”) and the ultimate (“why”) reasons of such a mechanism will impact on our knowledge of PCD in unicellular parasites and may provide clues for prospective drug targets.

A summary of studies on PCD markers in *P. falciparum* is presented in Table 1. On face value, the conflicting data offered by these studies seem daunting when attempting to reach a conclusion on PCD in *P. falciparum*. Consideration is thus given to possible explanations for the discrepancies that may lessen the controversy, and thereby either answer pertinent questions or raise new issues about the cell-death mechanisms of *P. falciparum*. This consideration is addressed in two parts. First, by considering confounding variables of the system, such as differences in the strain, stimulus, or life stage studied. Second, methodological pitfalls that may distort the interpretation of results are examined. By evaluating conflicting evidence as individual pieces of the same puzzle, rather than different pieces for the same open space, a larger and more descriptive picture forms.

## 3. Conflict due to Confounding Variables: Is There Method in the Madness?

### 3.1. *P. Falciparum* Strains

Numerous strains of *P. falciparum* have been used to study PCD, including chloroquine-resistant strains such as 7G8, FCR3, Lili, K1, PSS1, and Dd2, as well as strains that are sensitive to the drug, for example, 3D7 and F32 (Table 1). This complicates interpretation of the results when chloroquine (8 out of 12 studies) and other drugs are used to induce cell death, especially since it has been speculated that the development of chloroquine drug resistance may be correlated with a decreased susceptibility to undergo PCD [26]. Recently, reduced sensitivity to artemisinin has been described [40], but gene expression studies have not linked this phenotype to changes in PCD [41]. Different strains seem to differ in their susceptibility to undergo PCD and manifest different phenotypes. The 3D7 strain appears to be most susceptible to PCD with an apoptotic phenotype resulting from exposure of parasite cultures to chloroquine [12, 26, 34] etoposide [26], or increased temperatures [27]. Apoptosis has also been reported in the Dd2 strain under high *in vitro* parasite densities [36]. Other strains manifest entirely different cell death phenotypes, such as the PSS1 strain that showed evidence of autophagic cell death, as indicated by cytoplasmic vacuolisation without chromatin condensation or DNA fragmentation and caspase involvement [37]. The CSC-1 strain lacked markers of apoptosis and instead showed swelling and food vacuole lysis, resulting in secondary necrosis after stimulation with drugs or febrile temperatures [38]. Drug-induced cell death of the F32 strain did not exhibit any of the typical markers of apoptosis although the occurrence of PCD was not ruled out [25]. Conflicting results exist for the 7G8 strain, where chloroquine either induced apoptosis [26, 34] or caused cell death showing features of both apoptosis and autophagy [24]. Studies utilising multiple *P. falciparum* strains subjected to the same stimuli within the same laboratory provide the best evidence to show interstrain differences in PCD phenotypes and susceptibilities [12, 26, 34].

### 3.2. Cell-Death Stimulus

Widely differing stimuli (Table 1), concentrations, and exposure times have been used to study PCD in *P. falciparum, *which makes direct comparisons problematic. Antimalaria drugs are the most commonly used experimental trigger, but results have not been consistent. Treatment of cultures for 6 and 24 hours with 40 nM chloroquine (corresponding to the IC_50_ value of the drug) revealed DNA fragmentation suggestive of apoptosis [12]. However, another study using the same strain and stimulus showed almost no response at a comparable chloroquine concentration of 30 nM, with a significant effect only evident above 30 *μ*M chloroquine treatment for 8 or 10 hours [34]. Apart from this dose-dependent effect of a single drug, the mode of action of a drug also impacts significantly on the type of cell death. Nyakeriga and coworkers treated the F32 *P. falciparum* strain with several drugs with different modes of action and demonstrated that the effect on numerous PCD markers varied significantly [25].

A question arises as to whether antimalaria drugs are an appropriate stimulus, since the reaction of the parasite to the drug may be different in the *in vitro* system compared to the *in vivo* disease. In PCD experiments, the dose of the drug is adjusted so that the death rate is less than 100%, which correlates with the concept of PCD, where some parasites die to benefit the rest of the population although it may also reflect the fact that not all parasites have taken up the drug. However, *in vivo*, when a patient with malaria is treated, the drugs rapidly and effectively kill all parasites, implying that at least in some parasites, death is an uncontrolled event as opposed to a preprogrammed mechanism. Physiologically relevant triggers may therefore better reflect the pathways that operate *in vivo*.

During the intraerythrocytic cycle of development, *P. falciparum* is exposed to temperatures up to 41°C during periodic bouts of fever in the human host, which occur in response to erythrocyte rupture and release of new merozoites. Incubation of parasites at febrile temperatures has been used as a natural stimulus to study PCD but with conflicting results. One study showed DNA fragmentation after 2 hours at 41°C [27], whereas a similar study demonstrated no effect after incubation at 40°C for as long as 16 hours [38], albeit with different strains (3D7 and CSC-1, resp.).

**Figure 1:**
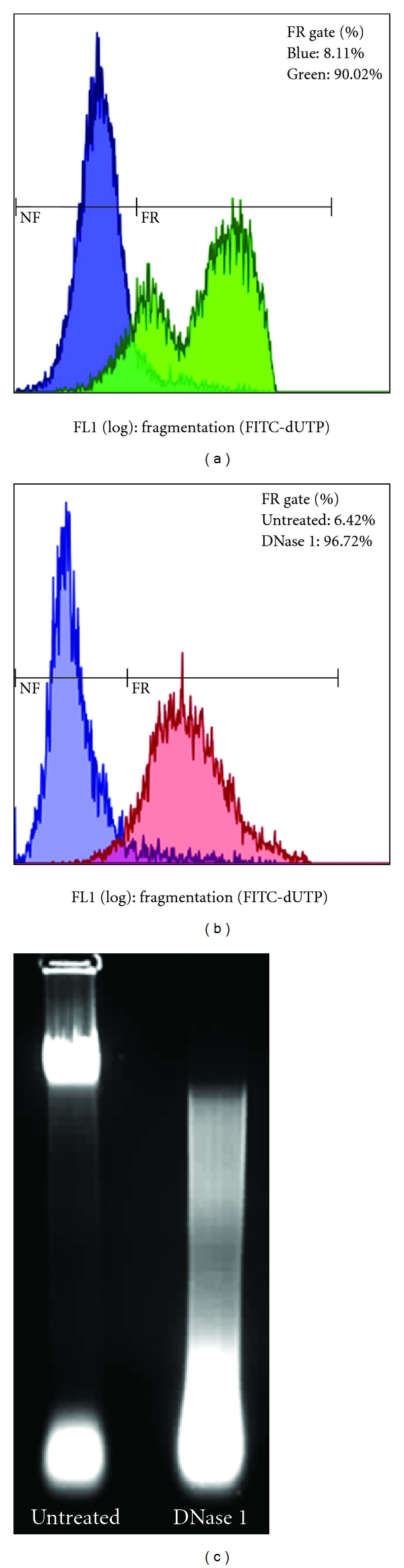
DNA fragmentation as illustrated by the TUNEL assay and agarose gel electrophoresis. Plot regions are denoted as nonfragmented (NF) and fragmented (FR). (a) TUNEL results showing parasites at 5.0% parasitaemia (blue) and parasites at parasitaemia of 7.2%, which failed to progress beyond the ring stage (green), after a decline from a high parasitaemia of >11%. (b) TUNEL results of untreated parasites (blue) and parasites treated with DNase 1 (red). (c) Agarose gel electrophoresis of genomic DNA from intact untreated (left) and DNase 1-treated (right) parasites showing a smear of DNA fragments.

The self-limiting effect of increasing population densities has also been investigated as a stressor for triggering PCD [36]. The Dd2 strain showed growth stagnation at <6% parasitaemia in highly synchronous cultures, with apoptotic markers including mild DNA fragmentation as measured by the TUNEL assay [36]. This contrasts with our studies on the 3D7 strain, which reached peak parasitaemia levels of >11% and following decline, exhibited very high levels of DNA fragmentation, also measured by the TUNEL assay (Figure 1(a)). These parasites remained in the ring stage of the life cycle and failed to progress to trophozoites and schizonts.

### 3.3. Life Cycle Stages and Culture Conditions

*P. falciparum* has a complex life cycle and is subjected to very different environmental conditions as it shuttles between its human and mosquito hosts. Evidence relating to PCD of the ookinete stage in the mosquito midgut was briefly mentioned earlier. Subsequent to invasion of human erythrocytes, the parasite develops through sequential stages of proliferation, which respond differently to external stimuli that may trigger PCD. This hampers the generation of consistent results, since researchers have used either asynchronous cultures or else different stages of synchronous cultures (rings, trophozoites, or schizonts). In addition, there is wide variation in initial parasitaemia and haematocrit as well as the composition of the culture medium (e.g., the use of human plasma or serum or different concentrations of Albumax).

Another variable in *in vitro* culture experiments relates to the presence of residual white blood cells (leukocytes) despite wash steps to purify erythrocytes. These cells may undergo PCD in response to the stimulus applied to induce parasite death, and since they contain DNA, this may interfere with studies of DNA fragmentation. This dilemma was illustrated by Porter et al. [38], who demonstrated oligonucleosomal DNA laddering by agarose gel electrophoresis in parasitised erythrocyte cultures treated with chloroquine, similar to that reported by Picot et al. [12]. However, the laddering pattern was absent once cultures had been depleted of leukocytes by affinity chromatography. Moreover, treatment of whole blood with chloroquine produced a similar laddering pattern [38]. Cognisance should therefore be taken of possible false positive results when interpreting DNA laddering or fragmentation experiments.

Finally, each stage of development is associated with a different repertoire of mRNA and proteins, which also confounds the issue and may offer another explanation of discrepancies in results, since the appropriate executors of PCD may not be present in the experimental stage under investigation.

## 4. Conflict due to Methodological Choice or Design: Madness in the Methods?

Apart from discrepancies in data due to differences in the studied system, as described above, it is also possible that problems may be encountered in the methods used to study the system. Morphological markers are unreliable although the cellular and molecular methods that have been recommended to replace them [28] are currently also not perfect and suffer from diverse drawbacks [42]. Technological approaches to detect markers of PCD in protozoan parasites are borrowed from studies in metazoans, and commercial kits have typically been optimised for mammalian systems. They may therefore not be appropriate for studies in *Plasmodium*. Some of the most common markers of PCD, especially apoptosis, that have been used in *P. falciparum* are DNA fragmentation, alterations in the mitochondrial and plasma membranes and the involvement of proteases. Some of the methodological pitfalls associated with these techniques are considered below.

### 4.1. DNA Fragmentation: Agarose Gel Electrophoresis versus TUNEL Assay

Oligonucleosomal DNA fragmentation is considered one of the hallmarks of the late stages of apoptosis, resulting from internucleosomal DNA cleavage, which produces a characteristic ladder of DNA fragments (see Table 2 in Appendix A) [2, 28]. Two methods of detection are used in *P. falciparum* studies: (1) agarose gel electrophoresis involving the electrophoretic resolution and visualisation of isolated DNA by ethidium bromide or SYBR green staining, or more commonly (2) the TUNEL (Terminal deoxynucleotidyltransferase-mediated dUTP Nick End Labelling) assay, which relies on enzyme (TdT)-mediated integration of a fluorochrome-conjugated base (dUTP) to free 3′-OH ends of fragmented DNA strands in whole cells, detected by flow cytometry, fluorescence microscopy, or light microscopy with appropriate filters [2, 43]. Table 1 provides an overview of the DNA fragmentation results obtained in various *P. falciparum* PCD studies. These results are occasionally in conflict with other detected markers of PCD in the same study. This may be due to the choice or execution of the detection method, as considered below.

The initial suggestion of PCD in *P. falciparum* was based on oligonucleosomal DNA fragmentation demonstrated by a ladder of DNA fragments resolved on agarose gels. The low sensitivity of conventional ethidium bromide staining necessitated radiolabelling of free DNA ends prior to electrophoresis and Southern blotting [12]. Our own data attest to the problem of low sensitivity: despite significant growth inhibition and the prominent appearance of crisis forms in chloroquine- and heat-treated 3D7 parasites, we have been unable to demonstrate the expected oligonucleosomal laddering pattern on agarose gels (1-2 *μ*g DNA loaded) (Engelbrecht et al., unpublished data). This contrasts with the findings of Ch'ng and colleagues who demonstrated DNA fragmentation by the TUNEL assay at comparable concentrations of chloroquine, using the same strain [34].

The TUNEL assay analysed by flow cytometry appears to be accepted as the standard for detecting DNA fragmentation in *P. falciparum.* However, the major drawback of this method is that it cannot distinguish the type of DNA fragmentation, and therefore, oligonucleosomal DNA fragments, as well as random fragments, will yield positive results. No definite conclusion on the type of PCD can thus be reached, since fragmented DNA with liberated 3′-OH ends may be generated in cells undergoing apoptosis, necrosis, or autophagy [43–46]. Our own results confirmed this shortcoming: *in situ* DNA digestion in *P. falciparum* with DNase 1, which is utilised in several studies as a positive control [25–27, 36, 37], showed ~90% TUNEL positive parasites (Figure 1(b)) although this treatment manifested as a homogenous smear of DNA (that could be associated with necrosis) on agarose gels stained with ethidium bromide (Figure 1(c)). A careful choice of controls for both apoptosis and necrosis is thus essential for correct interpretation of DNA fragmentation results. Perhaps exploitation of the ability of flow cytometry to measure several parameters simultaneously in individual cells may also provide the opportunity to include additional markers to discriminate between DNA fragmentation in apoptotic and necrotic cells.

Furthermore, analysis of the TUNEL assay by fluorescence microscopy [26, 27] is only semiquantitative and may not provide an accurate representation of the portion of the parasite population manifesting DNA fragmentation. These results should therefore be corroborated by quantitative flow cytometry. Totino et al. [37], observed <10% of TUNEL positive parasites using flow cytometry, despite significant (~40%–75%) parasite death, after treatment with staurosporine (an inducer of apoptosis), or SNAP (a nitric oxide donor) or the antimalaria drug, chloroquine. The authors thus suggested that TUNEL positive fragmentation, detected solely by fluorescence microscopy [26], may only represent a small fraction of the population [37], and since a small percentage of TUNEL positive parasites have also been observed in untreated cultures [34, 37], these results should be interpreted with caution. It should be noted, however, that Totino and coworkers assessed parasite viability with rhodamine staining, which correlates with an early apoptotic loss of mitochondrial membrane potential, whereas DNA degradation is a later event, and this may partly explain the lack of correlation between DNA fragmentation and parasite death [37].

With each method exhibiting its own drawbacks, one may ask which one, if any, of the above should be used to detect DNA fragmentation? Agarose gel electrophoresis is simple and economical to perform and provides conclusive evidence for the type of DNA fragmentation but suffers from low sensitivity and is only semiquantitative. The low sensitivity may be overcome by substantial, time-consuming modifications, such as Southern blot analysis [12]. The TUNEL assay, in contrast, is highly sensitive, rapid, and quantitative if assessed by flow cytometry. However, it cannot discriminate between specific oligonucleosomal cleavage of DNA and random degradation, which is essential in determining the type of cell death. It would thus be prudent to verify the type of DNA fragmentation with agarose gels for those samples that produce positive TUNEL results.

### 4.2. Loss of Mitochondrial Transmembrane Potential (Δ*ψ*_m_)

Mitochondria play a key role in PCD, and a loss of membrane potential (Δ*ψ*_m_) usually precedes complete permeabilisation of the mitochondrial membrane [28, 47], which triggers downstream events in the PCD cascade. In apoptosis, loss of Δ*ψ*_m_ is an early event occurring prior to other apoptotic manifestations such as chromatin condensation, DNA laddering, or phosphatidylserine (PS) externalisation [47]. However, decreased Δ*ψ*_m_ and mitochondrial membrane permeabilisation may also occur during early necrosis [28, 47, 48] prior to the appearance of vacuolisation and cytoplasmic swelling [47].

The mitochondrial membrane potential is normally evaluated with lipophilic cationic probes [47], such as DiOC_6_ [24, 25, 38], JC-1 [26, 34, 35], TMRE (tetramethylrhodamine ethyl ester) [36], or rhodamine 123 [37]. The fluorescent probes are detected either by spectrofluorimetry, flow cytometry, or fluorescence microscopy. As summarised in Table 1, a decrease in Δ*ψ*_m_ was observed after cell death had been induced in various strains with drugs or heat or bilirubin, but due to the nonspecificity of this parameter, it was not always correlated with apoptosis. Conflicting results were obtained by Nyakeriga and colleagues, who could not detect any loss of Δ*ψ*_m_ after treatment of the chloroquine-sensitive F32 strain with chloroquine or etoposide, a topoisomerase II inhibitor [25]. As expected, atovaquone, which targets the mitochondria, decreased the Δ*ψ*_m_ [25]. The choice of mitochondrial probe may have a significant influence on results, as probes differ in both their specific and nonspecific binding targets and are often mitochondrial inhibitors themselves [49, 50]. Various probes have also shown different responses depending on the stimulus used to induce mitochondrial dysregulation, albeit in a human cell line [50]. It is also crucial to ensure proper staining of the parasites and to include appropriate experimental controls. In addition to an untreated parasite culture and a nonparasitised erythrocyte control to monitor nonspecific binding, it is important to include a positive control, such as a mitochondrial uncoupling agent (CCCP), to ensure the validity of the results. Furthermore, the mitochondrial localisation of the dye should be verified with fluorescence microscopy. In view of the ambiguous nature of this marker, it cannot be used in isolation to determine the phenotype of PCD.

### 4.3. Phosphatidylserine (PS) Externalisation

Viable erythrocytes normally maintain an asymmetrical transbilayer distribution of phospholipids, with the anionic aminophospholipid phosphatidylserine (PS) localised almost exclusively on the inner leaflet of the plasma membrane [51]. In a number of conditions, such as sickle cell anaemia and thalassaemia as well as senescent erythrocytes, the asymmetry of the plasma membrane is lost, leading to externalisation of PS to the outer leaflet [51].

During apoptosis, complete mitochondrial membrane permeabilisation in response to a PCD stimulus results in the release of cytochrome c and calcium, which triggers the translocation of PS [28, 52]. This early PS externalization is a widely used marker of apoptosis in mammalian cells and has also been used in unicellular lineages [42]. In mammalian cells, the externalised PS is thought to serve as a signal to phagocytes to engulf and digest apoptotic cellular remnants, thereby preventing an immune response although the phenomenon has yet to be fully explained or exclusively linked to apoptosis [2, 53]. This flip-flop of PS is commonly detected with fluorochrome-labelled Annexin V, which binds to PS with high affinity although it may also bind to other anionic phospholipids [53]. The assay usually includes a membrane-impermeable vital dye such as propidium iodide to exclude demised cells that have become permeable.

**Figure 2:**
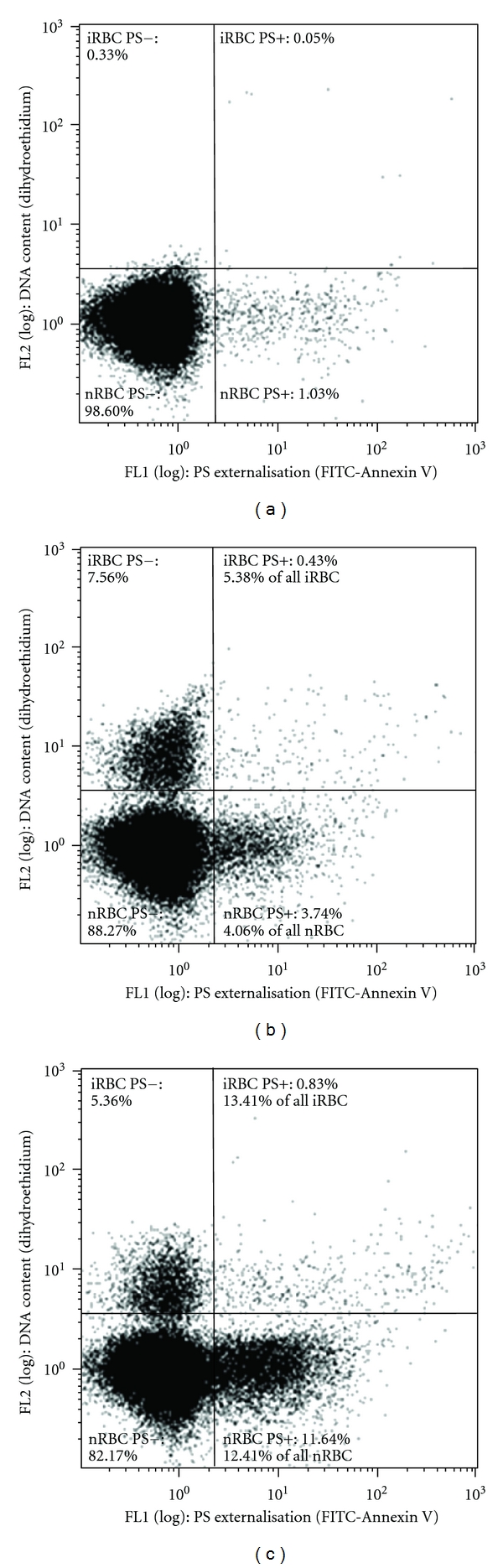
Phosphatidylserine externalisation (PS+) measured by FITC-Annexin V binding in both infected erythrocytes (iRBC) and their neighbouring noninfected erythrocytes (nRBC). iRBC were identified by the uptake of the cell-permeable DNA-binding dye dihydroethidium. An increase in PS externalisation can be seen by comparing an uninfected erythrocyte culture (a), to a parasite culture (b), both maintained at 37°C, and a heat-stressed parasite culture maintained at 40°C for 48 hours (c).

In *Plasmodium*, PS externalization has been detected during apoptosis of the extracellular ookinetes and zygotes of *P. berghei* [29, 30, 33]. However, detection of PS exposure in the intracellular erythrocytic stages is complicated by the presence of several membranes. Apart from the erythrocyte membrane, the parasite has a plasma membrane and is surrounded by a parasitophorous vacuolar membrane (PVM). The host cell membrane may be removed by extensive affinity purification steps and selective lysis of the PVM may be achieved with increasing concentrations of saponin or sorbitol [23, 54] although the purity of the parasite plasma membrane would have to be verified. The functional relevance of potential PS translocation to the outer surface of one of the parasite membranes is not clear, since the parasite is still within the intracellular milieu of the erythrocyte. However, *P. falciparum* remodels the host erythrocyte membrane extensively during its intracellular development, and although it initially prevents PS externalization to protect the infected host cell from clearance by macrophages, PS exposure becomes apparent during the schizont stage [6, 55, 56]. It is not known whether this change is specifically induced by a parasite undergoing PCD, but phagocytosis of the dead parasite will limit the immune response and production of inflammatory cytokines, which represents a survival advantage for the parasite. An investigation of the exposure of PS on the outer erythrocyte membrane surface after stimulating PCD in *P. falciparum *and a comparison to PS levels in untreated infected cells (Figure 2) should be correlated with other markers of PCD in the parasite.

### 4.4. Cysteine Protease Involvement

Apoptosis is a genetically regulated catabolic process, which is executed by a proteolytic cascade of cysteine proteases, known as caspases [28]. Thus far, no true caspases have been identified in *Plasmodium *[23] although plant-like metacaspases have been found [57]. Nevertheless, the activation of caspase-like enzymes during PCD has often been used as a marker for apoptosis as summarised in Table 1.

Detection of caspase-like activity in *P. falciparum* relies on the use of fluorochrome-conjugated substrates or inhibitors which emit a fluorescent signal after proteolytic cleavage of the peptide substrate (such as VAD or DEVD) or irreversible binding of the inhibitor to the enzyme. In addition, substrate analogues linked to fluoromethyl ketone have also been used as inhibitors. The emitted fluorescence is quantified by flow cytometry or spectrofluorimetry and/or visualization by fluorescence microscopy.

Increased caspase-like activity was demonstrated in response to chloroquine [34], staurosporine [34], and bilirubin [35]. However, other groups found no evidence of cysteine protease involvement after treatment with various drugs [37, 38] or heat [38]. During chloroquine-induced PCD, Ch'ng and coworkers used an array of inhibitors and concluded that clan CA cysteine proteases such as cathepsins and calpains mediated parasite death [34]. This is in contrast to studies implicating clan CD proteases (metacaspases) in response to chloroquine treatment [26] or caspase 3-like enzymes following bilirubin exposure [35].

These conflicting results highlight several important caveats. (1) Commercial kits and reagents have primarily been developed for mammalian systems, and *Plasmodium* enzymes may exhibit different substrate specificity. (2) Broad spectrum caspase inhibitors also inhibit other cysteine proteases [58], and off-target effects may also complicate the interpretation of results [59]. (3) The enzymes implicated by substrate and/or inhibitor assays in *P. falciparum* have not been characterised and conclusively linked to parasite apoptosis. (4) Caspase-independent apoptosis has also been described [60–62], and this may occur when proteases have been inhibited. Thus, the inhibitors may not prevent cell demise but simply shift the phenotype of cell death to one that is not dependent on the affected protease [63].

## 5. Is There a Cure for the Madness?

The bulk of the evidence presented in this paper favors the occurrence of some form of PCD in *P. falciparum*; however, the major debate centres around the phenotype. The current classification of distinct cell death phenotypes [28] is important, since a uniform nomenclature provides clarity to the field; however, it may also exacerbate the problem, since it is becoming evident that parasite death may in some instances display features and markers of more than one phenotype or possibly early and late characteristics of a single phenotype. This may relate to different subsets of parasites in the study population responding in a slightly different way to the death stimulus or initiating PCD at slightly different time intervals if cultures are not tightly synchronised, since some stages may be more susceptible to a specific trigger. The NCCD has recognised this dilemma and proposes that the move away from morphological features to biochemical characteristics may alleviate the situation [28].

To facilitate interstudy comparison and consistency, an integrated strategy whereby researchers evaluate as many appropriate markers as possible using standardised methods and culture conditions in a single study may be beneficial to resolve the current inconsistencies in the data. In addition, a more rigorous experimental approach will increase the confidence in the data. Appropriate controls are therefore essential, and an adequate number of technical and biological replicates should be assessed to ensure a reliable and consistent outcome.

## 6. Genomic Evidence of PCD in *Plasmodium*

*Plasmodium* lacks true caspases [23, 64, 65] but genomic evidence for metacaspases (see Table 3 in Appendix B) has been found in *P. falciparum *[23, 26, 32, 57, 66, 67], *P. berghei* [32], and *P. vivax* [32, 66] as well as *P. knowlesi and P. yoelii* [66].

These metacaspases belong to the C14 family of clan CD cysteine proteases. PfMC1 (PF13_0289) has been partly characterised using computational methods [26] and contains an N-terminal caspase recruitment domain (CARD) and a C-terminal catalytic domain including the histidine-cysteine dyad essential for activity. *P. berghei *parasites have been genetically manipulated to produce a knockout clone lacking the orthologous PbMC1 (PB001074) gene in an attempt to evaluate the role of the enzyme in apoptosis of sexual stage parasites [32]. A comparison of wild-type and knockout parasites has produced conflicting results. The gene either seems to be functionally redundant, since it had no effect on apoptotic markers [32] (unpublished data cited in [42]), or it appeared to modulate ookinete numbers [68].

Transcription data revealed that PbMC1 is actively expressed in all mosquito stages of *P. berghei* and in female gametocytes but not in the asexual erythrocytic stages of development. PfMC1 is transcribed in the blood stages of *P. falciparum. *However, transcription does not guarantee active expression of the protein, and thus, the involvement of metacaspases in cell death processes is still unresolved.

 A *P. falciparum* gene (PF10450c) coding for a putative apoptosis-related protein showed increased mRNA expression in parasites exposed to bilirubin, and this correlated with several other markers of apoptosis [35].

Genomic evidence [66] for the existence of elements of a PCD pathway in *P. falciparum* is presented in Table 3 of Appendix B. These genes encode proteins that are involved in all stages of PCD, including induction, regulation, and execution, and although it remains to be proven experimentally, these findings suggest that a classical PCD pathway exists in *Plasmodium*. However, the possibility that these proteins/domains have unrelated pleiotropic functions cannot be excluded. The identification of amino acid sequences with structural similarity to p53 DNA-binding domains in *P. falciparum* is a particularly exciting finding. The low sequence similarity between *Plasmodium* and known p53 DBDs and the evidence for p53-like activity in plants and green algae [69, 70] raise the intriguing possibility that p53-dependant apoptosis is extremely ancient or arose more than once in eukaryote evolution.

## 7. What Is the Role of PCD in *P. falciparum*?

Despite conflicting evidence presented in the previous sections, it seems clear that markers of PCD can be detected in *P. falciparum *in response to numerous stimuli. This raises the question of the relevance of this phenomenon in a protozoan parasite. Although the adaptive advantage of PCD is more easily understood in multicellular organisms, where the sacrifice of some cells contributes to the development and maintenance of the higher-level organisms [14], the idea of suicide is difficult to reconcile with unicellular parasites (reviewed in [1, 14]). It is generally argued that PCD in *P. falciparum* may have developed by group-level selection, providing a survival benefit to kin in a population of closely related individuals [23]. In *P. falciparum*, PCD may provide a number of benefits, and one of the most widely suggested is the self-limitation of the parasite's burden on the host, to facilitate transmission. However, it should not be assumed that the existence of a PCD mechanism in *P. falciparum* is an adaptive trait even if its execution provides a survival benefit to kin, population or species.

Empirical evidence for adaptive programmed death in unicellular lineages has only been provided in two organisms so far. In the yeast, *S. cerevisiae*, a genetically encoded altruistic aging programme has been demonstrated [71, 72], and in the green alga *C. reinhardtii*, the substances released during PCD were shown to benefit others, suggesting the phenomenon is a group level adaptation in this organism [73]. However, PCD may have evolved and been maintained as a nonadaptive or pleiotropic trait in other organisms and still be genetically regulated [14]. Furthermore, the lessons learnt from other unicellular organisms, including protozoan parasites, such as *Trypanosomes* or *Toxoplasma*, may not always apply to *P. falciparum, *especially to the highly virulent intraerythrocytic stages, which are under very different environmental constraints and pressure.

## 8. Can We Exploit PCD as a Novel Drug Target?

If PCD is a group-level parasite survival mechanism, it would have to be downregulated in a therapeutic context. In addition, PCD in *Plasmodium* would have to be adaptive in its nature in order for its manipulation by drug therapy to come at a significant survival cost to parasite populations.

To minimise harmful side effects in patients, a protein in the PCD pathway that is targeted should be sufficiently different from the human counterpart. Metacaspases have been implicated as PCD effector molecules in *P. falciparum,* and these enzymes are only found in protozoa. However, treatment of mice with a pan-caspase inhibitor, z-VAD, did not protect them from the lethal effects of experimentally induced cerebral malaria. A similar antiapoptotic therapeutic approach using transgenic mice that overexpressed Bcl-2 also failed [74].

An interesting aspect that has not been dealt with in this paper is that *Plasmodium *induces apoptosis in numerous human host cell types, such as endothelial, neuronal, and retinal cells [6]. In contrast, infected hepatocytes and erythrocytes are protected from apoptosis by the parasite to ensure its own survival [55]. An antiapoptotic therapy that uses erythropoietin as a neuroprotective adjuvant to prevent neuronal apoptosis provides a new treatment option. It has been successful in experimental murine cerebral malaria [75], and a recent human study showed that African children with high levels of erythropoietin were protected against neurological sequelae of cerebral malaria [76].

Since malaria is an acute and aggressive infection that requires immediate treatment with drugs that rapidly and effectively kill all parasites, the concept of treating patients with agents that promote PCD may not have the required effect unless they are used in the context of an adjuvant. However, future elucidation of PCD pathways may yield potential target proteins that are multifunctional, which will broaden the scope of the drug and interfere with more than one aspect of the parasite's biology.

## 9. Perspective

With the controversy surrounding PCD phenotypes in *P. falciparum* being further complicated by methodological difficulties in the detection of several PCD markers and the often ambiguous nature of the markers themselves, a more innovative approach may be required to successfully characterise and exploit possible PCD pathways in *P. falciparum*.

Current thinking on PCD in *P. falciparum* is based on a multicellular PCD paradigm, which may not be appropriate for the unicellular malaria parasite, since it may differ in its ultimate and proximate reasons in reaching the same cellular endpoint. However, with our current lack of knowledge of PCD in *P. falciparum,* the metazoan template is a useful frame of reference and provides a starting point to identify homologous PCD genes and proteins in *Plasmodium* species. The development of new and more powerful computational algorithms to investigate the highly unusual *P. falciparum* genome and proteome will help direct future investigations. The identification of key genes and biochemical characterisation of the recombinant *P. falciparum* proteins will facilitate development of parasite-specific reagents, which will provide new tools and improve the specificity and sensitivity of marker assays. Genomic evidence of PCD machinery also paves the way for parasite gene manipulation and the generation of selective knockout parasites, which can then be used to assess the functional role of the gene in PCD.

Numerous challenges still exist for researchers in this field, and a few of these unanswered questions are delineated in Appendix C. The current phase of research is mainly descriptive, whereby markers of PCD are assessed and documented following *in vitro* exposure of *P. falciparum *cultures to adverse conditions. This provides indirect evidence of key participants in PCD, such as cysteine proteases although there is still no clear molecular link between the markers and the actual biochemical events in the parasite and the final phenotype. This should form the basis for the next exciting phase of study and the challenge for researchers will be to elucidate and clarify the phenotypic expression of PCD and to identify and characterise the pathway(s) that underlie this fundamental biological process in *P. falciparum*.

## Supplementary Material

In a recent study, six key proteins or domains were selected based on their involvement in the four main stages of the p53-dependent pathway: induction (ATM), initiation (p53), regulation (MDM2, CR6 and IAP) and execution (peptidase C14). Hidden Markov model (HMM) libraries were constructed (supplementary file which is available online at doi:10.1155/2012/646534, [66]). A detailed analysis of the four *Plasmodium* genomes was performed using these HMM libraries, as well as an array of computational approaches including standard homology methods, phylogenetics, structural models and a novel evolutionary rate-based alignment algorithm FIRE (Functional Inference using the Rates of Evolution), which was developed to identify homologous and analogous genes in organisms with unusual genomes, such as *P. falciparum*, and hence low sequence similarity [81]. Hits are listed in the table below with E-values in parentheses. Fifteen hits with negative E-values were retrieved from the HMM library for p53 DNA binding domains (DBD) and two of these are included in the table. These encode proteins containing predicted p53 DBD-like structural folds (antiparallel beta sheets with greek key topology) according to PlasmoDB v5.5 and positive FIRE scores. The FIRE algorithm predicts the function of a domain based upon similar evolutionary rates and produced scores of 0.71 and 0.68 for PFE1120w and PFE0325, respectively (FIRE scores greater than 0.6 are suggestive of similar functions).

## Figures and Tables

**Table 1:** Summary of PCD studies in *P. falciparum*. Symbols indicate the following: Δ—change observed (increase or decrease indicated by ↑ or ↓, resp.); **×**—not done. SNAP—S-nitroso-N-acetyl-penicillamine.

Phenotype	Stimulus	Strain	Morphological changes	DNA fragmentation	Mitochondrial membrane potential	Protease involvement	Ref
Apoptosis	Antimalaria drugs	HB3 K1	**Δ **Microscopy	**Δ↑** TUNEL	×	×	[23]
	Bilirubin	NF-54	**Δ** Fluorescence microscopy	×	**Δ↓**JC-1 Spectrofluorimetry and fluorescence microscopy	**Δ↑** Caspase assay, spectrofluorimetry, DEVD substrate, and DEVD inhibitor	[35]
			×	**Δ↑ **Laddering, Agarose gel and Southern blotting	×	×	[12]
		3D7	**Δ **Microscopy	**Δ↑** TUNEL by fluorescence microscopy	**Δ↓** JC-1 Fluorescence microscopy	**Δ** VAD inhibitor	[26]
	Chloroquine		×	**Δ↑** TUNEL by flow cytometry and fluorescence microscopy	**Δ↓** JC-1 Flow cytometry and fluorescence microscopy	**Δ↑** Caspase assay, flow cytometry and fluorescence microscopy, VAD substrate, VAD, FA, FF, LLL, CA-074, and E64d inhibitors	[34]
		7G8	**Δ** Microscopy	**Δ↑** TUNEL by fluorescence microscopy	**Δ↓** JC-1 fluorescence microscopy	NO CHANGE VAD inhibitor	[26]
		K1	×	×	**Δ↓** JC-1 flow cytometry	×	[34]
	Etoposide	3D7 7G8		**Δ↑** TUNEL by fluorescence microscopy		**Δ **VAD inhibitor NO CHANGE VAD inhibitor	[26]
	Chloroquine	HB3		**Δ↑** TUNEL			
	Oxidants	K1					
	Etoposide	HB3	**Δ **Microscopy	×	×	×	
	Starvation	K1					[23]
	Heat	3D7		**Δ↑** TUNEL by fluorescence microscopy			[27]
	Increased parasite density	Dd2		**Δ↑** TUNEL by flow cytometry and fluorescence microscopy	**Δ↓** TMRE flow cytometry	**Δ↑** protease mRNA and protein	[36]

Apoptosis/Autophagy	Chloroquine * S. nudum* extracts	7G8	**Δ **Microscopy, electron microscopy	**Δ↑** TUNEL by flow cytometry	**Δ↓** DiOC_6_ Flow cytometry	×	[24]

Autophagy	Chloroquine SNAP Staurosporine	PSS1	**Δ **Electron microscopy	NO CHANGE TUNEL by flow cytometry	**Δ↓** Rhodamine 123 Flow cytometry	NO CHANGE VAD inhibitor	[37]

Necrosis	Chloroquine Staurosporine Heat	CSC-1	**Δ **Microscopy, electron microscopy	NO CHANGE Agarose gel with SYBR Green staining	**Δ↓** DiOC_6_ Flow cytometry	**Δ↓**Caspase assay, Flow cytometry and fluorescence microscopy, VAD, LETD, LEHD, and AEVD inhibitors	[38]

Undefined	Chloroquine	Lili	×	NO CHANGE Agarose gel and Southern blotting	×		[12]
	Etoposide	F32	NO CHANGE Microscopy	NO CHANGE TUNEL by flow cytometry	NO CHANGE DiOC_6_ flow cytometry		
	Atovaquone				**Δ↓** DiOC_6_ flow cytometry	×	[25]
	SNAP		**Δ **Microscopy	**Δ↑ **Smear, Agarose gel with ethidium bromide staining			
	Artemisinin Ceramide Mefloquine	FCR3	NO CHANGE Electron microscopy	NO CHANGE TUNEL and agarose gels	×	NO CHANGE Caspase 3 assay	[39]
	Staurosporine	3D7	×	**Δ↑** TUNEL by flow cytometry and fluorescence microscopy	**Δ↓** JC-1 Flow cytometry	**Δ↑** Caspase assay, flow cytometry, and VAD substrate NO CHANGE VAD inhibitor	[34]

**Table 2:** 

Cell death phenotype	Morphology	Common biochemical markers
Apoptosis	(i) Decreased cellular volume (pyknosis) (ii) Rounding-up of cell	(i) Loss of mitochondrial membrane potential (ΔΨ_m_)
	(iii) Intact plasma membrane blebbing	(ii) Cysteine protease activation
	(iv) Chromatin condensation	(iii) Phosphatidylserine externalization
	(v) Nuclear fragmentation	(iv) Oligonucleosomal DNA fragmentation
	(vi) Apoptotic body formation	(v) Exclusion of cell-impermeable vital dyes in early stages
	(vii) Minor changes in cytoplasmic organelles	(vi) Increase in reactive oxidants

Autophagy	(i) Cytoplasmic vacuolization	(i) Starvation response
	(ii) Autophagic vesicles with double membranes	(ii) Induction of membrane rearrangement genes (e.g. ATG1 and ATG8) to form autophagosomes
	(iii) No chromatin condensation	(iii) Caspase 8 involvement

Necrosis	(i) Increased cellular volume (oncosis)	(i) Nonspecific DNA degradation
	(ii) Loss of integrity and rupture of plasma membrane	(ii) Inclusion of cell-impermeable vital dyes
	(iii) Swelling of cytoplasmic organelles	(iii) Loss of ΔΨ_m_

**Table 3:** 

Protein/domain with function	*P. falciparum*	*P. knowlesi*	*P. vivax*	*P. yoelii*
ATM (detection of DNA damage)	PF13_0072 (−2) PFD0690c (−2)	PKH_051590 (−2)	PVX_084530 (−5)	—

p53 DBD (initiator of PCD)	PFE1120w (−2) PFE0325w (−1)	—	—	—

MDM2/SWIB (negative regulation of p53 activity)	PF10_0167 (−1)	PKH_060130 (−1)	PVX_001730 (−2)	PY00201 (−1)

CR6 (negative regulation of cell cycle)	MAL7P1.212 (−3)	PKH_101090 (−1)	—	—

IAP (inhibitor of apoptosis)	PFE0985w (−2)	PKH_101280 (−3)	PVX_080265 (−4)	PY00703 (−1)

Peptidase C14 (execution of apoptosis)	PF13_0289 (−22) PF14_0160 (−1) PF14_0363 (−1)	PKH_111640 (−25) PKH_133100 (−2) PKH_126800 (−1)	PVX_114725 (−22) PVX_085640 (−2) PVX_118575 (−1)	— PY04718 (−2) PY00663 (−1)

Abbreviations: ATM: ataxia telangiectasia mutated; CR: cell regulator; DBD: DNA-binding domain; IAP: inhibitor of apoptosis protein; MDM2: murine double-minute 2; SWIB: swinged wings locus complex B; —: no orthologue detected.

## Appendices

### A. Characteristics of Programmed Cell Death (PCD) Phenotypes (Table 2)

PCD may be defined as any cell death process that results from the activation of an intrinsic cell death programme; that is, it is a genetically regulated sequential process. In recognition of the recommendations of the Nomenclature Committee on Cell Death (NCCD) in striving towards uniform nomenclature [28], apoptosis, and autophagy are considered phenotypes of PCD in *Plasmodium.* **Apoptosis** is associated with highly characteristic cellular and biochemical changes in response to detrimental external or internal stimuli, resulting in cell death [2]. **Autophagy** represents the sequestration of cellular material into autophagosomes for subsequent degradation and typically promotes survival, but under extreme or prolonged adverse conditions, the cell may die [77]. Necrosis, once viewed as an entirely uncontrolled manner of cell death, may also be a genetically regulated and energy-dependent form of cell death [78]. However, necrosis is a poorly defined phenotype in *Plasmodium* and in the absence of evidence for a regulated sequence of events it is considered a non-PCD form of cell death in *Plasmodium*. Each cell death phenotype is characterised by a distinctive morphology; however, the NCCD has recommended that morphological cell death definitions be replaced with more objective functional or biochemical criteria. The main morphological features of the different cell death phenotypes and some of the common markers for each are listed in the table below. It should be noted that phenotypes may not be exclusive and overlap may therefore occur between the markers. Any marker in isolation should thus not be considered proof of a particular PCD phenotype.

### B. Genomic Elements of a PCD Pathway in Four *Plasmodium* Genomes (Table 3)

Evidence has recently accumulated demonstrating the manifestation of one or more markers of PCD in the asexual blood stages of *P. falciparum *in response to various experimental stimuli to induce parasite death. In the sexual developmental stages within the mosquito host, features of apoptosis have also been observed in *P. berghei *and* P. falciparum*. However, the presence of these markers does not necessarily imply the existence of a complete pathway.

There is very little information concerning the genes and proteins involved in PCD in *Plasmodium*. Some of the reasons for this lack of data relate to the fact that the *P. falciparum* genome is a notoriously difficult genome to investigate. It is evolutionary distant from other taxa due to fast-evolving genes and is phylogenetically ancient and complex [79]; there are frequent low-complexity regions and other inserts in genes, and the nucleotide and codon biases render the methods for orthologue identification less sensitive and specific. Conventional homology methods have therefore yielded limited results, which is evident from the finding that ~60% of predicted proteins in the *P. falciparum* genome have no significant homology to known proteins [80].

In a recent study, six key proteins or domains were selected based on their involvement in the four main stages of the p53-dependent pathway: induction (ATM), initiation (p53), regulation (MDM2, CR6 and IAP) and execution (peptidase C14). Hidden Markov model (HMM) libraries were constructed (supplementary file which is available online at doi:10.1155/2012/646534, [66]) based on multiple sequence alignments performed with MAFFT [81] and edited with BioEdit [82]. Construction, calibration and implementation of HMMs were conducted with HMMER [83]. A detailed analysis of the four *Plasmodium* genomes was performed using these HMM libraries, as well as an array of computational approaches including standard homology methods, phylogenetics, structural models and a novel evolutionary rate-based alignment algorithm FIRE (Functional Inference using the Rates of Evolution), which was developed to identify homologous and analogous genes in organisms with unusual genomes, such as *P. falciparum*, and hence low sequence similarity [84]. Hits are listed in the table below with E-values in parentheses. Fifteen hits with negative E-values were retrieved from the HMM library for p53 DNA binding domains (DBD) and two of these are included in the table. These encode proteins containing predicted p53 DBD-like structural folds (antiparallel beta sheets with greek key topology) according to PlasmoDB v5.5 and positive FIRE scores. The FIRE algorithm predicts the function of a domain based upon similar evolutionary rates and produced scores of 0.71 and 0.68 for PFE1120w and PFE0325, respectively (FIRE scores greater than 0.6 are suggestive of similar functions).

### C. Missing Pieces of the Puzzle

Apart from the problematic aspects regarding experimental studies on PCD in *P. falciparum*, which are highlighted in this paper and require further research, numerous additional and challenging questions remain to be answered. A few of these are outlined below.

(1) Which Life-Cycle Stage of P. falciparum Is Most Susceptible to PCD? During its complex life cycle in two hosts and diverse tissues, some stages may be more prone to PCD than others to maximise parasite survival. Ookinetes in the mosquito and rapidly growing intraerythrocytic trophozoites and schizonts appear to be more susceptible to various stimuli, in contrast to early ring stages. In particular, ring stages are apparently not affected by febrile temperatures, and this has biological relevance, since periods of fever in the host occur during this developmental stage. Does the exposure of merozoites to host immune factors render these extracellular forms of the parasite vulnerable to PCD?

(2) What Makes Some Parasites within the Population More Susceptible to PCD? The concept of PCD being adaptive in unicellular organisms implies that only a subset of parasites is destined to commit suicide to benefit the remaining members of the population, but how are they selected? Natural infections of *P. falciparum* in endemic areas exhibit a high degree of genetic diversity due to multiple infectious mosquito bites, and since PCD should benefit genetically similar or identical kin, this may play a role in the selection process. In areas of low transmission or in *in vitro* studies, the parasites are clonal, implying that other selection criteria are utilised. These are not yet known, but they may relate to small differences in metabolic activity of individual parasites or to differences in the microenvironment.

(3) What Are the Suicide Signals Inducing PCD? Several clues are available from the pathogenesis of malaria, which imply that the signals may potentially be provided by the host, an individual parasite or a population of parasites. In malaria patients, anaemia is a major and often life-threatening consequence of infection, and to keep the host alive, the parasite may elicit PCD in response to host factors such as a decreased haemoglobin content or haematocrit and altered blood viscosity. High parasitaemia in patients and in *in vitro* cultures, or a large ookinete burden in the mosquito, intuitively constitutes a parasite-derived PCD signal to protect the host, and thereby ensure parasite survival. Are the signals positive or negative? If trophozoites or schizonts develop abnormally or are damaged by internal or external factors, does this trigger removal of these defective parasites? Alternatively, if a merozoite fails to invade an erythrocyte, does the lack of a receptor-ligand interaction trigger a default PCD pathway?

(4) Is There Communication between Parasites within a Population? For parasite-derived signals to be effective, a quorum sensing mechanism must exist to allow parasites to be in touch with each other, but the nature of this communication system in *P. falciparum* is currently unknown. Furthermore, since PCD involves killing a subset of parasites only, how do parasites receiving the same message ensure that only some of them react?
