# Supplementary material for: On Programmed Cell Death in Plasmodium falciparum: Status Quo
Source: J Trop Med. 2012 Jan 12;2012:646534. doi: 10.1155/2012/646534 (PMC3263642; doi:10.1155/2012/646534)
Supplement: Supplementary file 1 — In a recent study, six key proteins or domains were selected based on their involvement in the four main stages of the p53-dependent pathway: induction (ATM), initiation (p53), regulation (MDM2, CR6 and IAP) and execution (peptidase C14). Hidden Markov model (HMM) libraries were constructed (supplementary file which is available online at doi:10.1155/2012/646534, [66]). A detailed analysis of the four Plasmodium genomes was performed using these HMM libraries, as well as an array of computational approaches including standard homology methods, phylogenetics, structural models and a novel evolutionary rate-based alignment algorithm FIRE (Functional Inference using the Rates of Evolution), which was developed to identify homologous and analogous genes in organisms with unusual genomes, such as P. falciparum, and hence low sequence similarity [81]. Hits are listed in the table below with E-values in parentheses. Fifteen hits with negative E-values were retrieved from the HMM library for p53 DNA binding domains (DBD) and two of these are included in the table. These encode proteins containing predicted p53 DBD-like structural folds (antiparallel beta sheets with greek key topology) according to PlasmoDB v5.5 and positive FIRE scores. The FIRE algorithm predicts the function of a domain based upon similar evolutionary rates and produced scores of 0.71 and 0.68 for PFE1120w and PFE0325, respectively (FIRE scores greater than 0.6 are suggestive of similar functions). [file 646534.f1.pdf]

Six key proteins or domains were selected based on their involvement in the four main stages of the p53-dependent pathway: induction (ATM), initiation (p53), regulation (MDM2, CR6 and IAP) and execution (peptidase C14). Sequence data for the eight elements were retrieved from NCBI (www.ncbi.nlm.nih.gov/), EBI (www.ebi.ac.uk), InterPro (www.ebi.ac.uk/interpro), Pfam 23.0 (pfam.sanger.ac.uk), PlasmoDB v5.5 (www.plasmodb.org/), and the protein UniProt (www.uniprot.org/) databases (a file containing accession numbers and edited MSAs is available on request). Multiple sequence alignments for each domain were performed with MAFFT [81] (algorithm G-INS-i, blosum62 matrix, gap-opening penalty of 1.53 and gap-extension penalty of 0.123), edited with BioEdit [82] and used to construct hidden Markov model (HMM) libraries. During model building the algorithm does not completely weight sequences according to the degree of divergence (evolutionary distance). To minimise potential model bias therefore, an evolutionary diverse group of taxa was selected and where appropriate, consensus sequences of over-represented groups, such as mammals or insects, were used. Construction, calibration and implementation of HMMs were conducted with HMMER version 2.5 [83]. Models were generated with GS (global single), GM (global multiple), LS (local single) and LM (local multiple) algorithms to increase the sensitivity of the library by detecting single and multiple domains, as well as conserved regions within a single domain. The default Gerstein / Sonnhammer / Chothia (GSC) weighting algorithm was used during HMM building and the library was validated by scanning the following four diverse genomes and correctly identifying the relevant proteins: *H. sapiens*, *D. melanogaster*, *C. elegans* and *E. histolytica*. Complete annotated peptide sequences of these four genomes were downloaded from the UCSC Genome Bioinformatics site (http://genome.ucsc.edu) and the *E. histolytica* Genome Project database (http://www.tigr.org/tdb/e2k1/eha1/new.shtml) and used in the validation.
